# Synchronous primary esophageal squamous cell carcinoma and gastric adenocarcinoma: analysis of 41 cases treated in a single institution

**DOI:** 10.1038/srep13335

**Published:** 2015-08-20

**Authors:** Xuyan Li, Suiling Lin, Yuling Zhang, Hongbiao Wang

**Affiliations:** 1 Department of Internal Medicine, Cancer Hospital of Shantou University Medical College, Shantou, Guangdong, China; 2 Department of prevention and health care, Cancer Hospital of Shantou University Medical College, Shantou, Guangdong, China; 3 Department of quality control, Cancer Hospital of Shantou University Medical College, Shantou, Guangdong, China

## Abstract

The present study investigated the treatment and survival outcomes of patients with synchronous primary esophageal squamous cell carcinoma and gastric adenocarcinoma. The medical records of 10,783 patients with primary esophageal squamous cancer treated at our institution between 1995 and 2012 were retrospectively reviewed. Overall survival (OS) rates were calculated using the Kaplan–Meier method. The incidence was 0.38% (41/10,783). Of these 41 patients, 26 underwent curative surgery, ten received palliative chemotherapy or radiotherapy, and five received no treatment. The median OS of the surgery, palliative-therapy, and treatment-free groups was 17.1, 9.0, and 3.8 months, respectively. The 1-, 3-, 5-, and 10-year OS rates for the surgery group were 77%, 45%, 33%, and 19%, respectively. No significant differences in median OS were observed between the surgery group and the historical cohort of isolated esophageal cancer (n = 186) (17.1 vs. 21.0 months, *P* = 0.061) or isolated gastric cancer (n = 51) (17.1 vs. 28.9 months, *P* = 0.875), or between the palliative-therapy group and its corresponding historical cohort (n = 30) (9.0 vs. 8.3 months, *P* = 0.862). The survival outcomes of patients with synchronous primary esophageal squamous and gastric cancers were not worse than those of patients with isolated esophageal cancer or isolated gastric cancer.

Esophageal and gastric cancers share certain risk factors, including diet, low socioeconomic status, age, and alcohol and tobacco use^1^,^2^. The development of sophisticated detection methods has resulted in an increase in the incidence of synchronous esophageal and gastric cancer. Esophageal squamous cell carcinoma is one of the most common cancers and a frequent cause of cancer-related death in the Chaoshan region of Southern China. The high number of patients with esophageal squamous cell carcinoma is associated with an increased number of synchronous gastric cancers with a pathological subtype of adenocarcinoma. However, there are no treatment guidelines for patients with such double primary cancers. In our institution, potentially operable patients are encouraged to undergo surgery with curative intent, whereas inoperable patients receive palliative chemotherapy or radiotherapy or both. However, the efficacy of these treatments has not been assessed. In the present study, we performed a retrospective analysis of the treatment and survival outcomes of patients with synchronous primary esophageal squamous cancer and gastric adenocarcinoma.

## Patients and Methods

Consent for the use of clinical materials for research purposes was obtained from all patients, and the study was approved by the Ethics Committee of the Cancer Hospital of Shantou University Medical College, China. All experimental protocols were approved by the committee. Informed consent was obtained from all subjects before the start of treatment. All procedures were performed in compliance with approved guidelines.

Synchronous gastric cancer was defined as follows: gastric cancer was detected before surgery or diagnosed preoperatively by esophagogastroscopy or a barium meal examination, or identified during surgery for esophageal cancer, and it was pathologically proven as adenocarcinoma after surgery.

Patients with pathologically proven esophageal squamous cell carcinoma were screened for enrollment in the study. Inclusion criteria were: pathologically proven synchronous esophageal squamous cell carcinoma and gastric adenocarcinoma diagnosed between January 1995 and December 2012 with available medical records and follow-up data.

Information on patient age, sex, preoperative work-up, treatment, and follow-up was extracted from medical records. Patients’ stages were reviewed according to the 6^th^ TNM classification.

Three historical controls were used that included 186 esophageal squamous cancer and 51 gastric adenocarcinoma patients who underwent radical surgery between 2001 and 2002, and 30 advanced esophageal squamous cancer patients treated with palliative chemotherapy between 2008 and 2011.

Survival curves and rates were calculated using the Kaplan–Meier method. Differences in survival were assessed using the log-rank test. A *P* value < 0.05 indicated a significant difference. All *P* values were two-sided.

## Results

Of 10,783 patients diagnosed with primary esophageal squamous cancer between 1995 and 2012, 41 had associated gastric adenocarcinoma, representing an incidence of 0.38%. The last follow-up was on May 14^th^, 2014.

**Figure 1:**
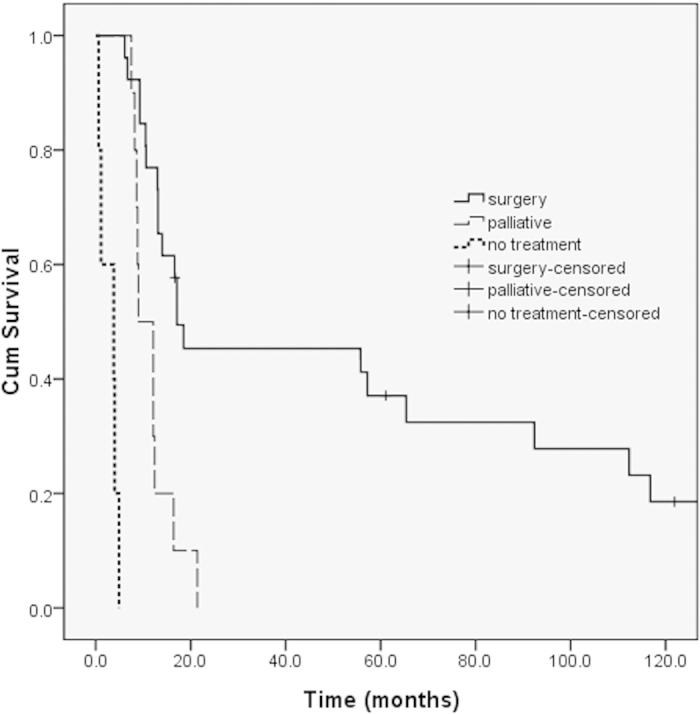
Survival curves for the surgery, palliative-therapy, and treatment-free groups.

The patients were divided into three groups based on treatment subtype as follows: 26 patients underwent curative surgery (surgery group), ten received palliative chemotherapy or radiotherapy (palliative-therapy group), and five received no treatment (treatment-free group). The median overall survival (OS) for the surgery, palliative-therapy, and treatment-free groups was 17.1, 9.0, and 3.8 months, respectively. Significant differences in median OS were observed among these three groups (*P* = 0.001 for the surgery group vs. the palliative-therapy group; *P* < 0.001 for the surgery group vs. the treatment-free group; *P* < 0.001 for the palliative-therapy group vs. the treatment-free group; Fig. 1)

### The surgery group

Of the 26 patients who underwent curative surgery, 21 were men and five were women, and four patients were alive at the time of the study. Two of the esophageal cancers were located in the upper, 13 in the middle, and 11 in the lower thoracic esophagus; 23 of the gastric cancers were located in the cardiac region, one in the fundus, and two in the corpus of the stomach (Table 1). There was no correlation between the esophageal cancer stage and the gastric cancer stage in these double cancer patients (*r* = 0.147, *P* = 0.416). Among the baseline characteristics of patients with esophageal cancer in the surgery group and the isolated esophageal cancer cohort, no differences in age, depth of invasion, and nodal status were observed. Significant differences in the location (*P* = 0.01) and grade (*P* = 0.02) were detected.

Esophagogastroscopy was performed in 26 patients, and 23 gastric adenocarcinomas were detected preoperatively. In one patient, severe stricture prevented passage of the endoscope into the esophagus; barium-meal examination indicated roughness in the cardiac mucosa. Gastric wall thickening was detected in two patients by computed tomography (CT).

Gastric tumors and esophageal tumors were removed simultaneously in all cases. Esophagectomy with lymph node dissection through thoracotomy and proximal gastrectomy with lymph node dissection were performed in 25 patients; the remaining stomach was used as an esophageal substitute. One patient underwent total gastrectomy using the jejunum for reconstruction. None of the patients died of postoperative complications.

The median age was 63 years (range, 45–74 years). The median OS was 17.1 months. The 1-, 3-, 5-, and 10-year OS rates for the surgery group were 77%, 45%, 33%, and 19%, respectively.

After surgery, 11 patients received no further treatment, 11 had cisplatin-based or 5-fluouracil-based adjuvant chemotherapy, four had adjuvant radiotherapy, and one had adjuvant cisplatin-based chemoradiation therapy. Adjuvant therapy was not associated with an overall survival benefit (hazard ratio = 1.85, 95% confidence interval: 0.76–4.49, *P* = 0.176).

Univariate analysis was performed in the surgery group to identify potential prognostic factors (age, sex, adjuvant chemotherapy, adjuvant radiotherapy, depth of invasion of esophageal or gastric cancer, nodal status of esophageal or gastric cancer, and stage of esophageal or gastric cancer) related to survival; however, no prognostic factors were identified.

**Figure 2:**
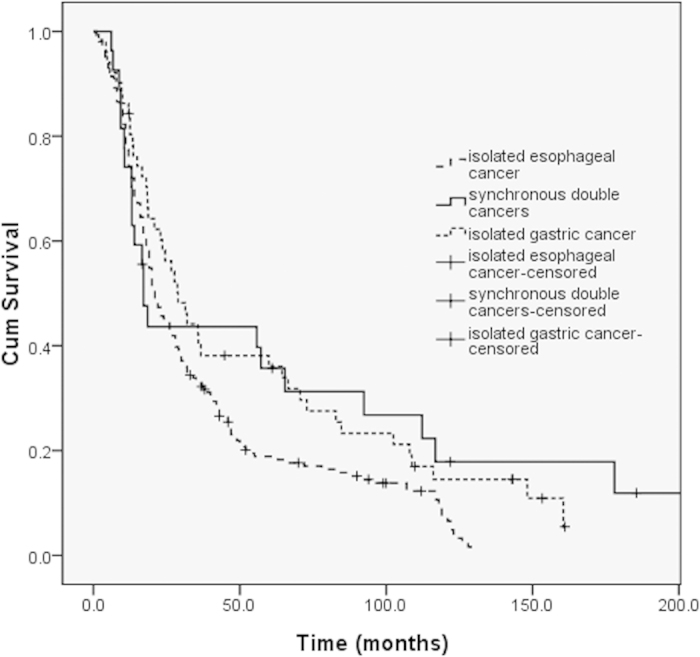
Survival curves of patients with synchronous double cancer, isolated esophageal cancer, and isolated gastric cancer.

A total of 186 patients with isolated esophageal squamous cancer and 51 patients with isolated gastric adenocarcinoma underwent radical surgery between 2001 and 2002 at our institution. In the isolated esophageal cancer group, 20 and 52 patients received adjuvant chemotherapy and radiotherapy, respectively. In the isolated gastric cancer group, 34 and three patients received adjuvant chemotherapy and radiotherapy, respectively. None of the patients received neoadjuvant therapy. No significant difference in median OS was observed between the surgery group and the isolated esophageal squamous cancer cohort (17.1 vs. 21.0 months, *P* = 0.061, Fig. 2), or the isolated gastric cancer group (17.1 vs. 28.9 months, *P* = 0.875, Fig. 2).

### The palliative-therapy group

There were ten men in this group with ECOG performance status of 0 or 1. All patients were diagnosed by endoscopy. Of these ten patients, six were diagnosed with metastatic disease; three of them were treated with 5-fluouracil-based chemotherapy and three received radiotherapy. Four patients who were considered ineligible for surgery because of invasion of adjacent tissues were treated with radiotherapy concurrently with cisplatin chemotherapy. None of the patients received second-line treatment. The median age was 68 years (range, 56–78 years). The median OS was 12.1 months, and the 1- and 2-year OS rates were 56% and 0%, respectively.

The historical control group consisted of 30 advanced esophageal squamous cancer patients who received palliative chemotherapy between 2008 and 2011. No significant difference in median OS was observed between the palliative-therapy group and the historical cohort (12.1 vs. 8.3 months, *P* = 0.862).

### The treatment-free group

There were four men and one woman in this group with ECOG performance status of 1, who refused to receive any treatment. Three patients presented with metastatic disease by CT scan, two with lung metastasis and one with liver metastasis. Two patients refused further examinations to define the clinical stage. The median age was 61 years (range, 51–65 years). The median OS was 3.8 months, and the 6-month OS was 0%.

## Discussion

The association of esophageal cancer with head and neck cancer has been demonstrated^3^,^4^,^5^,^6^,^7^; however, a high incidence of synchronous gastric cancer has also been reported, especially in East Asia^8^,^9^,^10^. In the present study, the incidence of synchronous primary esophageal squamous cell carcinoma and gastric adenocarcinoma was low compared to that reported previously, despite the large number of patients diagnosed with esophageal cancer in the Chaoshan region of Southern China. Most of the gastric lesions in this retrospective study were located in the upper third of the stomach (24 cases, 92.3%).

Because the stomach was used as an esophageal substitute in most patients with esophageal cancer treated surgically, the detection of gastric cancer was critical. Several techniques are available to detect gastric lesions. In cases of esophageal cancer in which severe stricture prevents the passage of the endoscope into the esophagus, the stomach should be carefully examined in a barium meal study or CT scan. Positron emission tomography with computed tomography (PET/CT) is the preferred technique to detect multiple lesions because of its high sensitivity^11^. Intraoperative gastric examination should be performed in cases in which gastric abnormalities are detected during the preoperative work-up.

Patients with multiple primary synchronous cancers are likely to show poor survival outcomes^12^,^13^. However, in the present study, the outcomes of patients with synchronous primary esophageal squamous cell carcinoma and gastric adenocarcinoma were not inferior to those of patients with isolated esophageal cancer, which is consistent with the results of a previous retrospective study^14^.

In the present study, the 26 operable patients who underwent radical surgery showed acceptable long-term survival rates compared with those of patients with isolated operable esophageal squamous cell or gastric carcinoma. Our results suggested that esophagogastrectomy is the appropriate treatment in non-advanced cases^15^; however, the role of adjuvant therapy remains unclear.

In ten inoperable patients, palliative chemotherapy or radiotherapy, without second-line treatment, resulted in a median overall survival of 9.0 months (range, 7.5–21.4 months). However, patients who received no treatment had a short survival time. Therefore, active palliative therapy should be recommended in patients showing good performance status.

The present study included the largest series of patients with synchronous primary esophageal squamous cell carcinoma and gastric adenocarcinoma analyzed to date. However, the present study had several limitations. First, data were collected retrospectively, which could lead to selection bias. Second, the data collected did not include all factors that could affect prognosis. Third, the findings of the study are not likely to affect clinical practice.

In conclusion, the possibility of gastric adenocarcinoma should be considered before a patient undergoes surgery for esophageal cancer. Early detection and treatment of these lesions in non-advanced patients may improve survival. Palliative therapy should be considered in advanced patients.

## Additional Information

**How to cite this article**: Li, X. *et al.* Synchronous primary esophageal squamous cell carcinoma and gastric adenocarcinoma: analysis of 41 cases treated in a single institution. *Sci. Rep.* **5**, 13335; doi: 10.1038/srep13335 (2015).

## Figures and Tables

**Table 1:** Characteristics of the surgery group.

Characteristics	Synchronous double cancers (%)		Isolated esophageal cancers (%)	Isolated gastric cancers (%)
	Esophageal cancers	Gastric cancers		
Age (median, range)	63 45–71		57 33–77	55 36–78
Male	21(80.8%)		139(73.7%)	31(60.8%)
Female	5(19.2%)		47(26.3%)	20(39.2%)
Location				
Upper third	2 (7.4%)	24 (92.3%)	21 (11.3%)	2 (3.9%)
Middle third	13 (50.0%)	2 (7.7%)	144 (77.4%)	12 (23.5%)
Lower third	11 (42.6%)	0 (0%)	21 (11.3%)	37 (72.6%)
Depth of invasion				
T1–2	8 (30.8%)	10 (38.5%)	41 (22.0%)	14 (27.4%)
T3–4	18 (69.2%)	16 (61.5%)	145 (78.0%)	37 (72.6%)
Lymph node metastasis				
Negative	11 (42.3%)	9 (34.6%)	87 (46.8%)	14 (27.4%)
Positive	15 (57.7%)	17 (65.4%)	99 (53.2%)	37 (72.6%)
Grade				
I	3 (9.6%)	4 (15.4%)	49 (26.3%)	2 (3.9%)
II	14 (53.8%)	9 (36.6%)	120 (64.5%)	10 (19.6%)
III	9 (36.6%)	13 (48.0%)	17 (9.2%)	39 (76.5%)
Total	26		186	51

## References

[b1] Mao W. M., Zheng W. H. & Ling Z. Q. Epidemiologic risk factors for esophageal cancer development. Asian Pac J Cancer Prev. 12, 2461–6 (2001). 22320939

[b2] de Martel C., Forman D. & Plummer M. Gastric cancer: epidemiology and risk factors. Gastroenterol Clin North Am. 42, 219–40 (2013). 23639638 10.1016/j.gtc.2013.01.003

[b3] Kumagai Y. *et al.* Multiple primary cancers associated with esophageal carcinoma. Surg Today. 31, 872–6 (2001). 11759880 10.1007/s005950170025

[b4] Shibuya H., Wakita T., Nakagawa T., Fukuda H. & Yasumoto M. The relation between an esophageal cancer and associated cancers in adjacent organs. Cancer. 76, 101–5 (1995). 8630859 10.1002/1097-0142(19950701)76:1<101::aid-cncr2820760115>3.0.co;2-d

[b5] Natsugoe S. *et al.* Synchronous and metachronous carcinomas of the esophagus and head and neck. Dis Esophagus. 10, 134–8 (1997). 9179485 10.1093/dote/10.2.134

[b6] Natsugoe S. *et al.* Multiple primary carcinomas with esophageal squamous cell cancer: clinicopathologic outcome. World J Surg. 29, 46–9 (2005). 15592914 10.1007/s00268-004-7525-y

[b7] Nagasawa S., Onda M., Sasajima K., Takubo K. & Miyashita M. Multiple primary malignant neoplasms in patients with esophageal cancer. Dis Esophagus. 13, 226–30 (2000). 11206637 10.1046/j.1442-2050.2000.00116.x

[b8] Koide N. *et al.* Synchronous gastric tumors associated with esophageal cancer: a retrospective study of twenty-four patients. Am J Gastroenterol. 93, 758–62 (1998). 9625123 10.1111/j.1572-0241.1998.220_a.x

[b9] Pasławski M., Złomaniec J., Rucińska E. & Kołtyś W. Synchronous primary esophageal and gastric cancers. Ann Univ Mariae Curie Sklodowska Med. 59, 406–10 (2004). 16146021

[b10] Hamabe Y. *et al.* Clinicopathological features of esophageal cancer simultaneously associated with gastric cancer. J Surg Oncol. 68, 179–82 (1998). 9701211 10.1002/(sici)1096-9098(199807)68:3<179::aid-jso9>3.0.co;2-2

[b11] Miyazaki T. *et al.* Effectiveness of FDG-PET in screening of synchronous cancer of other organs in patients with esophageal cancer. Anticancer Res. 34, 283–7 (2014). 24403475

[b12] Naomoto Y. *et al.* Multiple primary cancers of the esophagus and thyroid gland. Jpn J Clin Oncol. 29, 349–52 (1999). 10470660 10.1093/jjco/29.7.349

[b13] Bai Y., Zou D. W. & Li Z. S. Clinical presentation, endoscopic features, treatment and prognosis of synchronous upper gastrointestinal malignancies. J Dig Dis. 13, 19–23 (2012). 22188912 10.1111/j.1751-2980.2011.00548.x

[b14] Poon R. T., Law S. Y., Chu K. M., Branicki F. J. & Wong J. Multiple primary cancers in esophageal squamous cell carcinoma: incidence and implications. Ann Thorac Surg. 65, 1529–34 (1998). 9647053 10.1016/s0003-4975(98)00177-5

[b15] Polotsky B. E., Machaladze Z. O. & al-Ansary N. M. Some aspects of multiple esophageal and gastric cancer. Semin Surg Oncol. 8, 46–9 (1992). 1589686

